# Evidence that ferritin is associated with light production in the mucus of the marine worm *Chaetopterus*

**DOI:** 10.1038/srep36854

**Published:** 2016-11-10

**Authors:** Renu Rawat, Dimitri D. Deheyn

**Affiliations:** 1 Scripps Institution of Oceanography, Marine Biology Research Division, University of California San Diego, 9500 Gillman Drive, La Jolla, CA 92093, USA

## Abstract

The blue glow of the mucus from *Chaetopterus* involves a photoprotein, iron and flavins. Identity and respective role of these components remain, however, largely unresolved today, likely because of viscosity issues and inhibition of this system by oxidizers conventionally used to track bioluminescence activity. Here, we used gentle centrifugation to obtain a mucus supernatant showing no inhibition to oxidizers, allowing for further analysis. We applied conventional chromatographic techniques to isolate major proteins associated with light emission. Luminescence ability of elutriate fractions was tested with hydrogen peroxide to track photoprotein and/or protein-bound chromophore. Fractions producing light contained few major proteins, one with similarity to ferritin. Addition to the mucus of elements with inhibitory/potentiary effect on ferritin ferroxidase activity induced corresponding changes in light production, emphasizing the possible role of ferritin in the worm bioluminescence. DNA of the protein was cloned, sequenced, and expressed, confirming its identity to a *Chaetopterus* Ferritin (ChF). Both ferric and ferrous iron were found in the mucus, indicating the occurrence of both oxidase and reductase activity. Biochemical analysis showed ChF has strong ferroxidase activity, which could be a source of biological iron and catalytic energy for the worm bioluminescence when coupled to a reduction process with flavins.

The marine worm *Chaetopterus sp*. (Annelida, Polychaeta), also known as the parchment tubeworm, lives in U-shaped tubes buried in sand/mud from canyons of continental slopes to shallow waters of coastal regions around the World^1^,^2^. The tubes are open at both ends to allow water-flow that the worm generates for feeding on detritic particles. The worm (adult size up to 10 cm long; Fig. 1A) produces an abundant amount of mucus that could have several functions associated with tube building, food trapping, and/or defense against predation^3^,^4^,^5^. In that case, one can imagine that the glowing mucus would stick to the intruding predator, interfering with its normal locomotion while also making it more visible, and thus a more vulnerable target to its own predators.

The *Chaetopterus* worm has been the subject of studies on its bioluminescence, which is associated with the secretion (neurally-controlled) of a luminous mucus upon physical disturbance^2^,^6^. The mucus produces a blue glow that is initially very bright, viz. when still within the worm body parts (Fig. 1B,C); intensity of the glow then fades as the mucus is secreted, remaining visible with the naked-eye for minutes when in solution (DDD, pers. obs.). The biochemical reaction behind this light emission remains unknown today. It is reported to involve a photoprotein that is iron regulated^7^,^8^ and an inhibitor cofactor that remains to be identified^8^. The mucus contains fluorescent compounds like riboflavin and FMN, yet their relationship with the light producing mechanisms remains to be clarified as well^9^. A protein of molecular weight 120–130 kD that was isolated from whole worm tissues (not from the mucus only) was associated with light production property, but never sequenced or identified^8^. Since then not much information is available on the identity of proteins present in the luminous mucus.

The bioluminescence of *Chaetopterus* is affected by iron^7^,^8^. In the wild, it is possible that modulation of the light production relies on iron dissolved in seawater from the surrounding sandy/muddy environment where the worm lives. However, it is more likely that the mucus contains endogenous iron (which would be secreted with the mucus to modulate its light production), since the worm kept in artificial seawater (with no iron) can produce mucus with strong luminescence^7^. Any of these situations however infers that the mucus could contain iron-affinity proteins (such as siderophore and/or ferritin-like proteins) to adsorb/release iron during modulation of the luminous reaction.

Hydrogen peroxide is a strong inhibitor of light production from native (un-treated) viscous mucus, which could be related to changes in proteins cross-linking and further rheological thickening of the mucus^7^. Accordingly, whole worm extracts that were chemically treated for thinning mucus –thus turning into weakly viscous material- behave like conventional luminous systems in that they produced low spontaneous light, but could be stimulated to glow using hydrogen peroxide^8^.

Here, to identify proteins involved in the mucus bioluminescence, we aimed at separating the mucus into two phases, highly and weakly viscous, and continue the work only with the weakly viscous phase. However, instead of using chemical treatment for mucus thinning^8^, we instead gently centrifuged the secreted mucus to collect a weakly viscous supernatant, which behaved like conventional luminous systems. We then performed a partial purification of light producing proteins from this supernatant, using the combination of anion exchange, ultrafiltration, and size exclusion chromatography. The few fractions showing the ability to produce light contained only a few major proteins, one of which matching a ferritin-like protein. We report the purification technique, cDNA sequencing, and recombinant protein expression as well as first evaluation of iron affinity for this *Chaetopterus* Ferritin (ChF). We also provide biochemical evidences that ferritin is closely associated with light production in the mucus, and discuss the mechanisms by which this could take place.

## Results and Discussion

### *Chaetopterus* mucus contains glycosylated proteins

*Chaetopterus* native mucus is viscous and shows rheological properties similar to a yield-stress elastic gel material^7^, thus similar to mucus of other organisms. Here, we showed that the mucus contains carbohydrates as well as proteins (Table 1), in correlated concentrations (Fig. S1); this suggests the constitutive proteins of the mucus are glycosylated. Most abundant monosaccharides included glucose, galactose, and glucosamine, followed by ribose, mannose, fucose and galactosamine (Table 1). The protein and carbohydrates concentrations were different amongst body parts, being the greatest for the tail (holding the gonads, but also releasing luminous mucus) and the aliform parapodia (from where the most intense luminescence is produced). Although the luminescent properties of the mucus appear independent from its rheological properties^7^, the data presented here indicate that the light production process could involve glycosylated proteins/compounds.

### Light producing activity can be separated from the viscous material of the mucus

The luminous mucus can be separated into two phases: one phase has spontaneous light production that is inhibited by hydrogen peroxide, while the other phase has no spontaneous light production but can be stimulated to produce light by hydrogen peroxide. Gentle centrifugation (7,000 g, 10 min) of the native mucus was used to generate these two phases. Such gentle centrifugation is not intense enough to pellet down proteins (which requires ultracentrifugation) but can pellet down structures (lamellar bodies, lattices) formed from cross-linked glycosylated proteins such as mucins^10^. Such gentle centrifugation is in fact used to separate active compounds from the mucin network^11^,^12^, the active compounds then being “squeezed out” of the mucus and found in the less-viscous, and easier-to-work-with, supernatant. Here, the highly viscous phase (pellet) behaved like the native mucus in terms of bioluminescence, producing spontaneous light that was inhibited by H_2_O_2_ (Fig. 2). In contrast, the weakly viscous phase (supernatant) showed little spontaneous light, but the light production increased in intensity with H_2_O_2_, as opposed to be inhibited by it (Fig. 2). The light production occurred as a long-lasting glow with similar kinetics to that of the native mucus (Fig. 2), indicating they share strong similarities in reagents responsible for the light production.

### Light production relies on a protein but also on salt and smaller molecules

The luminous mucus was fractionated by column chromatography and tested along the process for chemiluminescence activity (CL), which was used to assess concentration of proteins related to the light production process (Table S1). As expected, there was a loss in the chemiluminescence activity (yield) with progress in the fractionation and purification process, which was associated particularly with low recovery of CL activity and protein purification after using the PD-10 desalting column (step 2), after ultrafiltration (step 4a) and after gel filtration (step 5) (Table S1). This indicates that elemental concentration and/or smaller molecules could contribute to optimal light production. The smaller molecules are likely not to include a chromophore because chemiluminescence activity was found in no other fractions than those considered here for further analysis.

Protein concentration (absorbance at 280 nm, A_280 nm_, for proteins in general, and absorbance at 360 nm, A_360 nm_, for Fe^3+^-loaded ferritin specifically) and chemiluminescence activity followed a similar elution profile, peaking between elution volume 92.5 mL to 100 mL (Fig. 3A). The one fraction with the highest CL activity also showed the maximum A_360 nm_ (typical of proteins with Fe^3+^-ferrihydrite complex^13^,^14^), while showing a dip at A_280 nm_. This indicates that proteins without a ferrihydrite complex (thus with 280 nm absorbance) are eluted when chemiluminescence activity was not detected. Hence, the light production probably involves a protein complex that is associated with a ferritin-like structure containing Fe^3+^.

HiTrapQ fractions with high CL activity and high A_360 nm_ were pooled together, concentrated (by ultrafiltration on 9 kD MWCO kit; MWCO: Molecular Weight Cut Off) in MilliQ water, and subjected to size exclusion chromatography on Superdex75 column. Eluted fractions were monitored and assayed for CL activity with H_2_O_2_, similarly to the method used for HiTrapQ fractions. The process resulted in co-elution of several proteins collected in the fractions between volume 48.0–50.0 mL; these fractions retained some CL activity. Protein peak concentration A_280 nm_ was offset compare to the CL activity, while the alignment was better with A_360 nm_ (Fig. 3B). There were clear correlations between CL activity and absorbance at both 280 nm and 360 nm, except for higher A_280 nm_ values where CL activity tapered off relative to the amount of proteins (Fig. 4). This suggests that additional proteins in these concentrated fractions were not necessarily -or directly- involved in the light production process, which was not observed for the A_360 nm_ which specifically relates to a protein with an iron core (ferrihydrite)^13^,^14^.

The luminous fractions always contained multiple proteins that co-eluted together (Fig. S2). These proteins could be constitutive of the viscous mucus and/or associated with control of the light production since no increase in fold purification was observed based on CL activity (Table S1). The luminous fractions showed no sign of viscosity, and because the amount of light is indicative of the amount of protein involved in the light production process^2^,^8^,^15^, we kept a particularly close attention to the most abundant proteins vs light production through the purification process.

An 8-fold purification with low protein quantity was observed in flow through fraction from Ultrafiltration by 9 k MWCO membrane filter (step 4b) indicating a possible loss of cofactors (low molecular weight compounds) in the filtrate and during size exclusion chromatography. The flow through fraction analyzed in a separate study^9^ was found to contain fluorescent compounds, riboflavin and FMN (Flavin Mono Nucleotide). This suggests that one of these flavin compounds could be a cofactor in addition to Fe^2+^, required in peroxide-mediated bioluminescence reaction as suggested earlier^8^. One possible explanation is that these small compounds are bound to the same protein by weak non-covalent bonds and are thus easily separated off from the protein, including under low levels of salts. Specifically here, this would mean a ferritin-like protein would have association with flavins. Alternatively more than one protein could be important for light emission, where one protein would manage iron as cofactor while the other protein would be a carrier-protein for the organic chromophore (e.g., flavin). However, these two proteins would have to be in close proximity for light production to take place, which is difficult to consider in a system that is secreted in the environment. Nonetheless, the point here is that the *Chaetopterus* photoprotein likely requires the presence of iron (ionic cofactor) and flavins (possible exogenous chromophore).

### A ferritin is abundant in the mucus of *Chaetopterus*

Three major proteins were consistently found together co-eluting from Superdex75 columns. The proteins were well separated on SDS-PAGE gel (Fig. S2), which allowed their analysis by LC-MS/MS (Liquid Chromatography-Mass Spectrometry). Data from the most abundant of these proteins (the most intense band at 19 kD) is presented here while analysis of the other proteins (12 and 25 kD) is still in progress. Sequencing of the 19 kD band resulted in three peptides, each with 99.7% significance hit to ferritin from other marine worms (Table 2). None of these worm species though are reported to have the ability to produce bioluminescence, while all live in tubes. *Pectinaria gouldii* (the ice cream cone worm), *P*. *koreni* (the trumpet worm) and *Capitella teleta* (the Capitella worm) are common on beach and salt marshes. The Capitella worm is particularly known because of its cosmopolitan distribution and its wide popularity amongst scientists due to its genome sequence available. *Alvinella pompejana* (the Pompeii worm) is distinct from the previous ones by being endemic to deep-sea hydrothermal vents. It is not surprising to have found ferritin in *Chaetopterus* considering the widespread distribution of this protein through organisms^16^,^17^,^18^, but it is unique however to have found it secreted with luminous mucus.

To confirm the secretory nature of the ferritin in *Chaetopterus*, we performed its full sequence after extraction of total RNA from the worm aliform parapodia. We then successfully PCR amplified a 519 bp DNA fragment (Fig. S3) using primers designed from the three peptides obtained from the 19 kD protein. The DNA fragment was cloned and sequenced in *E*. *coli*. A 519 bp full-length cDNA that encodes for a 172 amino acid *Chaetopterus* Ferritin (ChF) was sequenced and submitted to GenBank (Accession Number KF471029). ChF has an important N-glycosylation (GlcNAc) site NQSL (residues 109–112) which follows the consensus pattern N-x-[ST]^19^, suggesting it is likely glycosylated and secretory in nature, which would be in line with our findings made above on the carbohydrate analysis. Another conserved site identified in ChF was the Protein Kinase C phosphorylation site SIK (residues 142–144) in the C-terminus of ferritin sequence follows a consensus pattern [ST]-x-[RK]. Kinase C prefers serine or threonine residues close to C-terminal for phosphorylation^20^. N-Glycosylation and Kinase C site could be important in determining the primary structure of ChF and it’s role in the secretion of protein out of the cell by trans-membrane transport, as suggested for other ferritins^21^,^22^,^23^. This confirms ChF has the molecular signals to be secreted as constitutive part of the luminous mucus.

A_360 nm_ in the chromatographic fractions with higher CL activity corroborates with the presence of ferritin in luminous fractions. Ferritin shows absorption near 360 nm wavelength mainly due to its ferrihydrite (Fe^3+^ oxide) [Fe_2_O_3_.0.5H_2_O] core^13^,^14^. This is the first report of a ferritin found in fractions able to produce light. Whether the ferritin is involved directly or indirectly in the light production process remains to be determined. However, we described in the previous section a clear correlation between CL activity and level of A_360 nm_ thus suggesting a direct contribution of ferritin in the light production. This does not appear coincidental since the bioluminescence in *Chaetopterus* uses iron as a cofactor^7^,^8^, and as discussed in the previous section. Thus having an iron-regulatory protein such as ferritin in the luminous mucus could be related to the control mechanisms of the light production (see last section).

### Both Fe^2+^ and Fe^3+^ occur in the *Chaetopterus* mucus, in abundance related to the amount of bioluminescence produced

The mucus secreted by the worm contained significant amount of iron present in the oxidized ferric form, but also the reduced ferrous form (Fe^2+^; 2.41 10^−5^ to 8.76 × 10^−3 ^mM with the ferrozine assay) accounting for about <10% to about 50% of the total iron (Table 3; Fig. 5). Since iron naturally oxidizes in water to its more stable ferric form, this indicates the mucus contains a protein capable of keeping ferrous iron from oxidizing, or a biochemical system able to reduce back some of the ferric iron. Such concentration of iron in the mucus demonstrates that the iron must originate mainly from the worm tissues, with limited contribution from external (environmental) sources. The measurements were highly variable from one replicate to the other (Table 3), which is likely related to the biology and life history of the worm. Such a specimen-variability was indeed reported earlier in reference to other biochemical analyses^7^,^8^. However, the general trend was that iron concentration (both Fe_tot_ and Fe^2+^) increased with light intensity from the mucus (Fig. 5), supporting the fact that iron is a native cofactor of the bioluminescence in *Chaetopterus*.

As expected iron was found in different body parts of the worm, in concentration ranging from 13.11 to 1,630 × 10^−3 ^mg/g (Fig. S4), thus being about 2 to 200× more concentrated than in the mucus (compare to Fe_tot_; Table 3). The iron concentration covered similar ranges amongst body parts although it tended to be greater for the black and white sacks. The mid/gut section, the cup-shaped parapodia and the tail contained the least amount of iron (Fig. S4). This range of iron concentration in tissues is about 3×–5× greater than levels found in a coastal scavenging marine polychaete in pristine areas^24^ while similar to levels found in polychaetes from coastal contaminated areas^25^. Our values are in fact in the same range as these measured for *Capitella*^26^ as well as for *Pectinaria* and one specimen of *Chaetopterus*^27^.

### Ferroxidase activity of *Chaetopterus* ferritin could have a role in light production

*Chaetopterus* ferritin sequence resembles Heavy chain (H chain) vertebrate ferritin and has iron(II) binding region, which imparts ferroxidase activity to catalyze the conversion of ferrous (Fe^2+^) to ferric (Fe^3+^) ions. The ferroxidase activity for the recombinant ferritin was 22.8 nmoles/min/mg. The kinetics parameters used to assess the affinity of the ferritin for iron were V_max_ = 0.81 ± 0.03 μM/min, and K_m_ = 0.016 mM (Fig. 6). This ChF K_m_ value is greater than the one usually reported for human ferritins that are commercially available. However, it is otherwise within the range identified for human ferritins when considering the ones used in research settings, which show K_m_ values ranging from 0.0006 to 2.5 mM^28^,^29^,^30^,^31^,^32^.

Assuming that the ferritin ferroxidase activity affects light production, we considered the mucus light production a proxy of the level of ferroxidase activity, thus indicating that the greater the light production, the greater the ferroxidase activity (and vice versa). To verify this, we analyzed the mucus light production when treated with trace elements known to affect (positively or negatively) the ferroxidase activity of ferritin. Monovalent elements (Cs, Li, Rb) had no significant effect on the mucus light production (Fig. 7A), which suggests no effect on the ferritin ferroxidase activity; this is indeed the case since ferritin reacts mainly with di- and tri-valent ions^33^. The only exception comes from Rb that has been shown to slow down iron-loading in the ferritin core compare to other mono-valent ions^34^.

Divalent elements tended to be inhibitory (Cd, Ni, Co, Cu, Zn, Mn), although only significantly for Co (72.2 ± 9.3%; P = 0.0406), Zn (74.7 ± 15.7%; P = 0.0335) and Mn (35.3 ± 9.6; P = 0.0010) (Fig. 7A). This is consistent with the inhibitory effect these elements have on ferroxidase activity^35^,^36^,^37^,^38^; Cu however is usually reported to enhance the ferroxidase activity^39^. As for Ca and Mg, they showed a potentiation of the light production, especially for Ca although not statistically significant (135.7 ± 30.5%; P = 0.2609). The effect of Ca on ferritin ferroxidase activity has indeed been report to increase this activity^40^, which would thus be consistent with our data when looking at the light production output (Fig. 7A).

Trivalent elements also showed potentiary (La) or inhibitory response (Al, Cr) that were all significant, for La (144.1 ± 44.5; P = 0.0455), Al (74.4 ± 14.5; P = 0.0161) and Cr (74.9 ± 14.6l; P = 0.0310) (Fig. 7A). Interestingly, La is usually reported to stimulate ferritin ferroxidase activity^41^ while Al reported to decrease it^42^,^43^. In general for all elements tested having an effect, the inhibition was initiated right away after injection of the element (e.g., for Mn), while the potentiation (e.g., for La) was more gradual (Fig. 7B). Overall for all the tested elements, there is a consistent correlation between the effect these elements have on ferroxidase activity and the corresponding effect on the mucus light production. These data provide strong evidence that ChF is closely involved in the mechanisms leading to light production in the worm mucus.

A strong ferroxidase activity would be in agreement with the fact that the worm relies on iron for its biology, including its ability to produce and/or control its light production. Indeed, ferrous iron was reported to be a cofactor in the light production, possibly involving the Fenton chemistry^2^,^7^,^8^. However, the Fenton reaction is a rather fast molecular event that occurs as a by-product of metabolic reactions in all biological systems^44^,^45^,^46^. If indeed associated with light production, the Fenton reaction needs tight regulation to allow two situations: (a) not trigger production of light in tissues when not stimulated to do so (the worm does not show constant bioluminescence in the body), and (b) trigger production of light in a sustained manner when the mucus is secreted. Indeed, the light production in the mucus can possibly last more than 12 hours (Fig. 8), which cannot be sustained by the Fenton reaction alone if not controlled.

Ferritin could play this regulatory role since its ferroxidase activity can convert Fe^2+^ into Fe^3+^, thus preventing the Fenton reaction to take place inside the cells [allowing situation (a); Fig. 9a] while storing the iron in a less-reactive form^47^,^48^. This scenario does not lead to light production. Once secreted, the Fe^3+^ of the ferritin can be reduced in the presence of reduced riboflavin, its derivatives (FMNH_2_, FADH_2_) and/or molecular oxygen, which are all present in the mucus^7^,^9^. This process allows a controlled release [allowing situation (b); Fig. 9b] of Fe^2+^ from the core of the ferritin^49^,^50^. This ferrous iron is reactive and there are three mechanisms through which it can be associated with light production (Fig. 9b), with still the possibility to also involve cofactors/inhibitors reported by other studies^8^,^9^,^51^,^52^. In one mechanism, the ferrous iron interacts directly with the worm photoprotein or chromophore, which likely induces allosteric changes and energy transfer leading to light production (Fig. 9b; luminescence b1). In another mechanism, the ferrous iron is oxidized by the molecular oxygen present in the mucus, which releases electrons then available to the worm photoprotein or chromophore for light production (Fig. 9b; luminescence b1). In a third scenario, the ferrous iron undergoes the Fenton reaction, thus producing hydroxy radicals and reactive oxygen species. Although the Fenton reaction itself produces yellow/orange luminescence at 575 nm^53^, its intensity is not strong enough to be detected in the luminous mucus^7^,^9^. In any case, it is commonly agreed that it is the reactive radical compounds associated with the Fenton reaction that are involved in the mechanism of intense light production^54^,^55^,^56^. The recipient molecules of these reactive radical compounds determine the spectrum of the luminescence, which can range from blue to red, as identified from *in vitro* chemical experiments^54^. In the case of the worm mucus, such recipient molecules can either be a photoprotein or chromophore, which would then be excited and able to produce light (Fig. 9b; luminescence b2). Such contribution of the Fenton reaction as a source of energy for the bioluminescence reaction is similar to what was proposed for other organisms^2^,^57^, occurring as long glows as for bacteria^58^,^59^, or rapid flashes in marine scale-worms^60^,^61^. In our case, the oxidation-reduction processes of the Fenton reaction would benefit from being coupled with the oxidation-reduction processes of the flavins that make ferrous iron available in the worm mucus. Such coordination would indeed make the light production last for long durations, which was evidenced by >15 hrs long lasting light production (Fig. 8), but also by the co-occurrence of both ferric and ferrous form of iron in the mucus (Table 3; Fig. 5). In addition, the fact that the light production depends mainly on oxidation-reduction processes allows the bioluminescence in the worm to be produced without the direct need of molecular oxygen, which was shown earlier^7^.

If the evidences are strong about the contribution of ferritin in the light production process, the identity of the photoprotein or protein-bound chromophore emitting the light still remains unclear. It could be the flavin like in bacteria^58^,^59^. Here, however, the flavin would be associated within a matrix that changes as the mucus gets spent, considering that the mucus shows the typical fluorescence of flavins only after exhaustion of the light production^9^. Work is in progress to identify the chemical nature of the chromophore complex.

## Conclusion

The luminous mucus from the marine worm *Chaetopterus* contains three major proteins, the most abundant one showing similarities to a ferritin. Identification of the worm ferritin (ChF) has been successfully achieved after purification, sequencing, cloning and expression of the recombinant ChF. Working with the mucus, we demonstrated the contribution of the ferritin in the light production process by showing a correlation between the inhibitory/activator effect of certain trace elements on the ferroxidase activity of ferritins, and the mucus light production. This is the first time a ferritin is found in association with bioluminescence. Although the exact functional relationship between the ferritin and the light production remains to be described, it could involve the storage of iron to facilitate the light production since this process is iron-dependent in the mucus. A controlled release of iron by the ferritin would indeed allow a long-lasting catalytic Fenton reaction, thus with a slow generation of peroxides that could be involved in light emission, possibly along with other cofactors. This study provides the first insights into the role of iron chemistry in the luminous mucus, and is an important step towards understanding the unique biochemical process of bioluminescence in *Chaetopterus*.

## Material and Methods

### Specimen collection

Bundles of parchment tubes of the *Chaetopterus* worm were collected from the La Jolla submarine canyon by SCUBA and kept in circulating ambient sea water in the Marine Biology Research Division Experimental Aquarium (Scripps Institution of Oceanography) until use, as previously described in details^7^.

### Partial purification of major proteins from the luminous mucus of *Chaetopterus*

**Figure 1:**
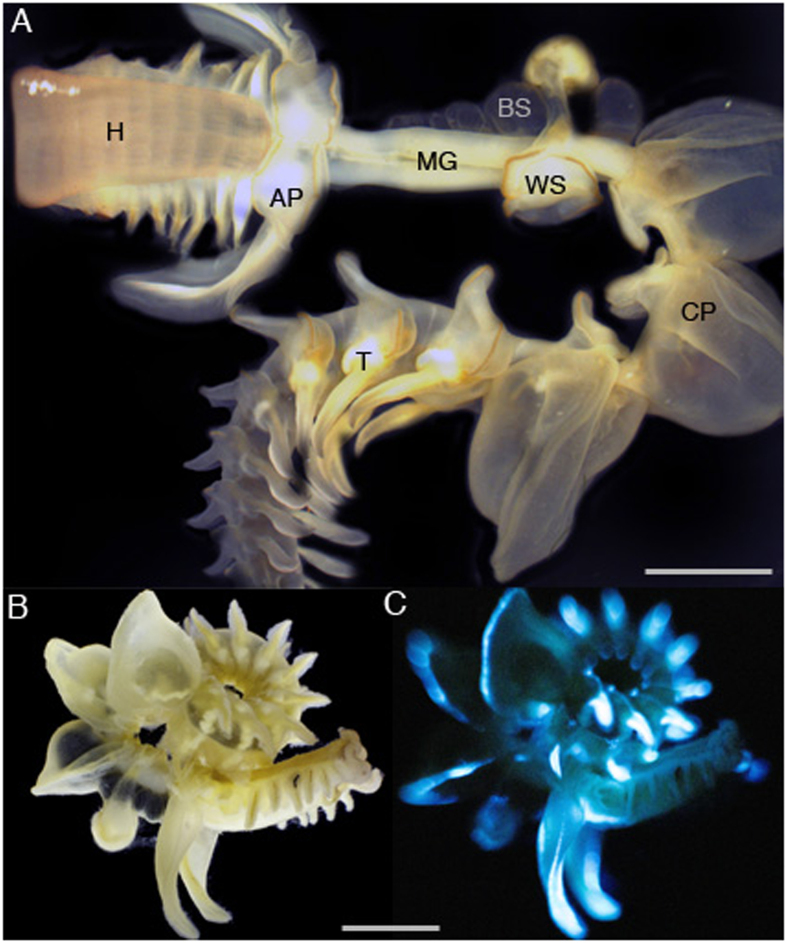
*Chaetopterus* sp. body parts (**A**) and view of the entire animal in bright field (**B**) and the corresponding view while producing blue bioluminescence (**C**), after addition of KCl 400 mM. Legend- H: Head; AP: Aliform Parapodia; MG: Mid/Gut section; BS: Black Sack; WS: White Sack; CP: Cup-shape Parapodia; T: Tail. Pictures 1B,C courtesy of David Liittschwager.

**Figure 2:**
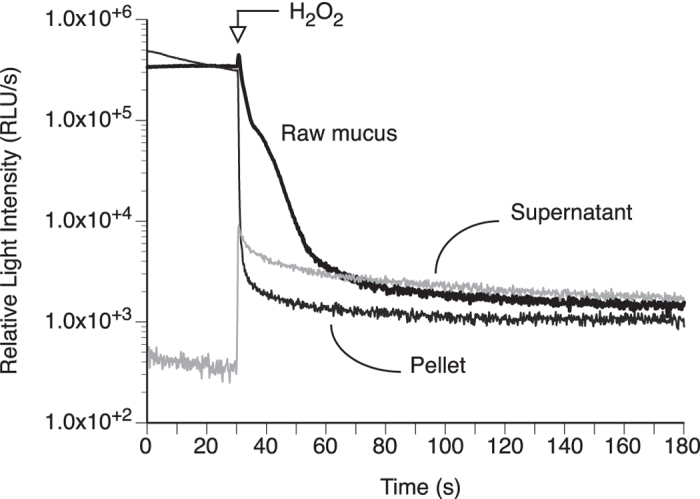
Representative kinetics of bioluminescence for the *Chaetopterus* mucus after gentle centrifugation. Light production was measured from native mucus, weakly viscous supernatant mucus and heavily viscous pellet mucus, before and after addition of H_2_O_2_.

**Figure 3:**
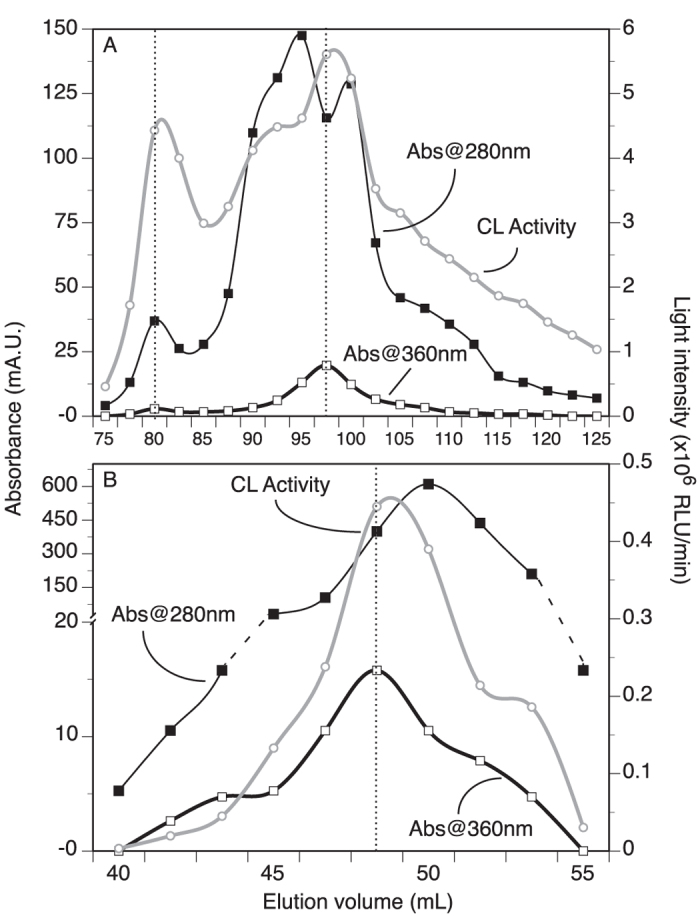
Elution profiles of the *Chaetopterus* luminous mucus following chromatography, and monitoring of CL activity (following addition of H_2_O_2_), A_280 nm_ (general protein) and A_360 nm_ (iron-core specific protein). (**A**) Using a HiTrapQ column, fractions with CL activity eluted around 80 mL (less specific front elution) but mainly between 92.5 and 100 mL. (**B**) Using a Superdex75 column with the most active fractions of the HiTrapQ run, only two fractions showed strong CL activity. In both elution profiles, peak fractions with CL activity corresponded better to A_360 nm_ peak fractions.

**Figure 4:**
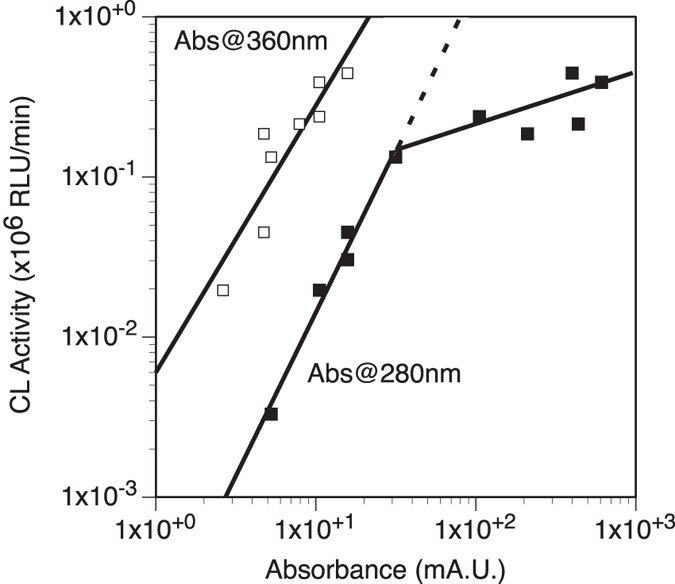
Correlation between absorbance and CL activity after the Superdex75 column purification step of the *Chaetopterus* luminous mucus. Correlation with A_360 nm_, representative of the iron-loaded ferritin, is preserved across all absorbance, whereas the correlation was not found for higher A_280 nm_ values (expected dotted line compare to filled squares at higher absorbance).

**Figure 5:**
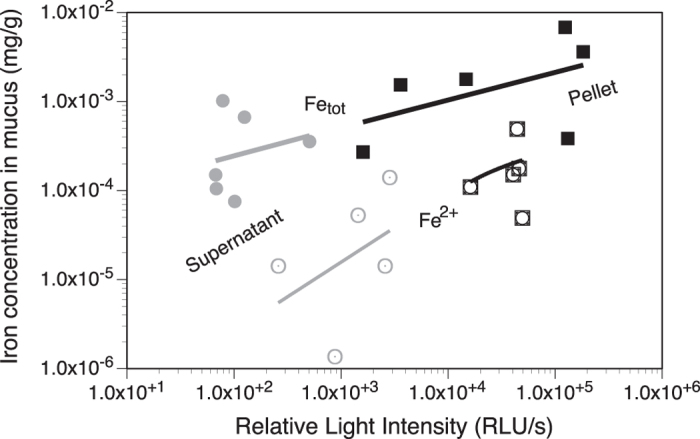
Concentrations of iron measured from the pellet and supernatant phases of the mucus obtained after gentle centrifugation, and expressed as a function of the initial intensity of light produced by each phase. Fe_tot_ was measured by ICP-OES, while Fe^2+^ by the Ferrozine assay.

**Figure 6:**
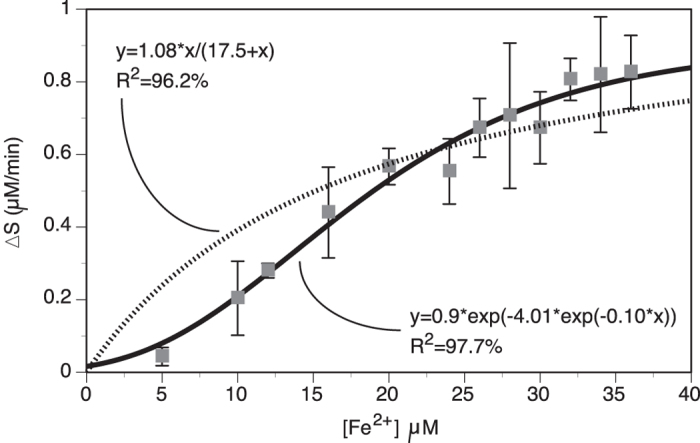
Ferroxidase activity of recombinant ferritin from *Chaetopterus*. Measurements were made in sodium-acetate buffer at pH 6.0 with varying concentration of Fe^2+^ using affinity-purified recombinant ferritin. Data (mean ± SE, N = 3) were plotted with nonlinear linear models, Michaelis-Menten (dotted line) and Gompertz (black line).

**Figure 7:**
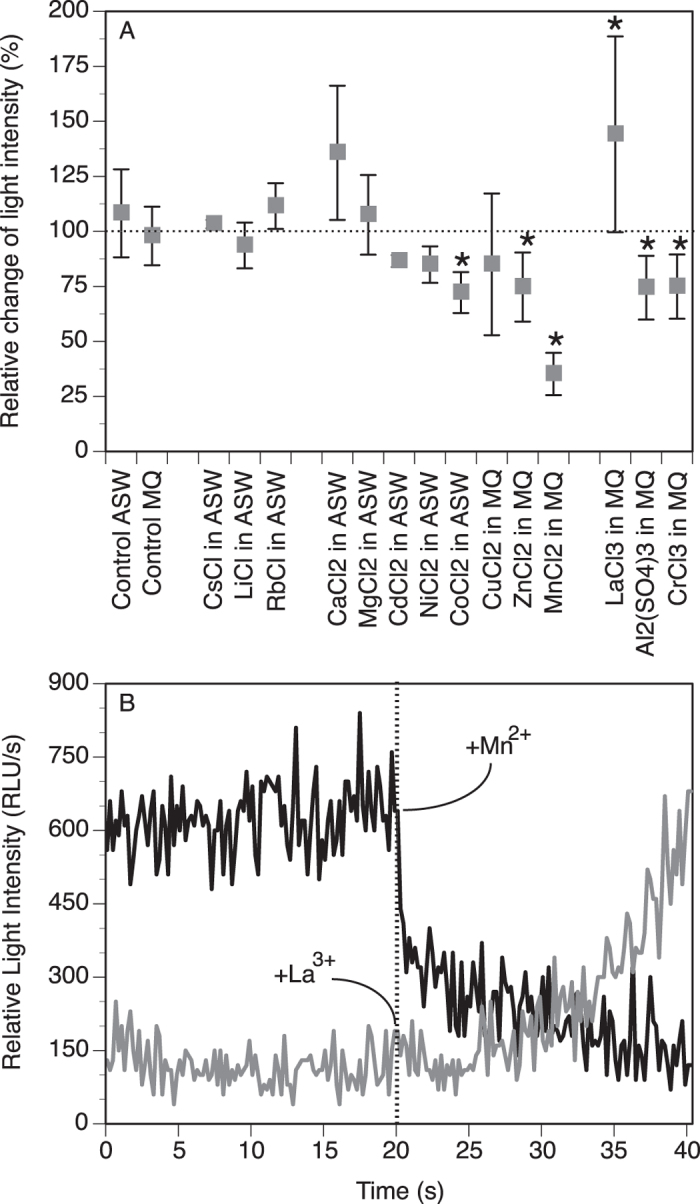
Effect of trace elements (mono-, di- and tri-valents) on the light production of the mucus supernatant. (**A**) Relative change (mean ± SD in %) after addition of the element compare to before addition. *Statistically different (P < 0.05) from the corresponding control with MQ or ASW only. (**B**) Representative kinetics of light production showing inhibition for Mn^2+^ and potentiation for La^3+^ when added at 20 s.

**Figure 8:**
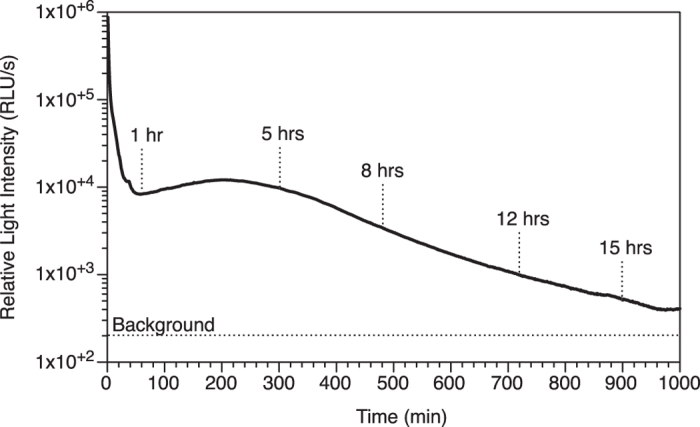
Representative kinetics of bioluminescence from the native mucus let to glow spontaneously over a long period of time (1,000 min).

**Figure 9:**
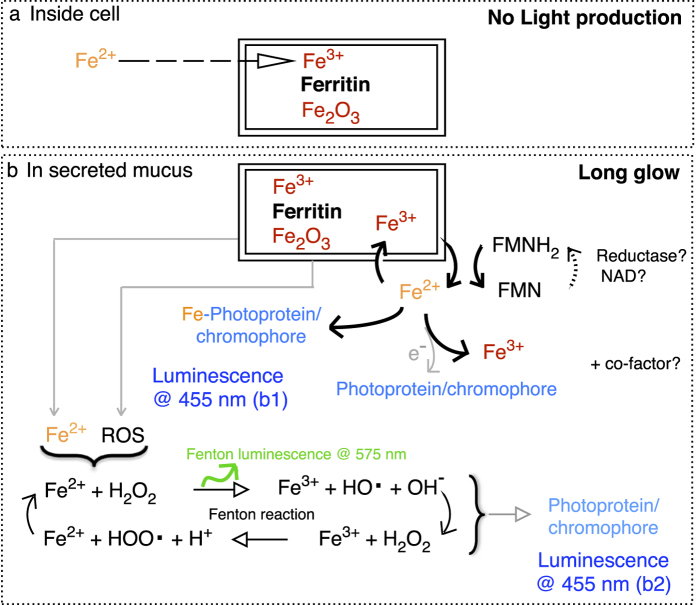
Schematic representation of the possible mechanisms that store iron inside cells when the worm does not produce light (**a**), and that release iron and reactive oxygen species (ROS) outside cells when the worm secretes the luminous mucus (**b**). The coupling of iron reduction with riboflavin oxidation can occur directly or via interaction with the worm photoprotein/chromophore (Luminescence b1) or together with the Fenton reaction (Luminescence b2). Flavin Mononucleotide can be oxidized (FMN) or reduced (FMNH_2_), through cycling with Nicotinamide Adenine Dinucleotide coenzyme (NAD).

After removal from the housing tube, each worm was cut using a scalpel to separate the anterior part from the posterior tail (Fig. 1A); the anterior part was used for further experiments while the tail was discarded because of possible gametes content. A total of 50 anterior parts were collected individually within a couple of hours, and the mucus collected from each separately by addition of 0.4 M KCl in Artificial Sea Water (ASW), which facilitates mucus secretion^7^. About 0.5 to 1 mL of KCl solution was used to allow the anterior part to be immersed. The treatment took place in individual Petri dishes on ice for 30 min, after which the viscous extract (then made of KCl solution and mucus mixed together) was collected using disposable pipets, and pooled together. A 50 μL aliquot of the pooled extract was tested for light production using a Sirius luminometer (Berthold Detection Systems, Germany) equipped with two automated injectors. The light production was measured for a total of 3 min, with v/v injection of 12% hydrogen peroxide (H_2_O_2_) at 30 s. The light intensity was recorded at a rate of 0.2 s and expressed in Relative Light Unit per second (RLU/s). The pooled extract was made clear of macroscopic chunks and debris by centrifugation at 7,000 g for 10 min at room temperature. This separated the pooled extract into a highly viscous pellet phase, and a weakly viscous supernatant phase. Both phases were tested for light production as described above; the supernatant phase was the only one considered for further analysis.

The supernatant phase was desalted in Q start buffer (50 mM Tris-HCl pH 7.9) using gravity flow 8.3 mL PD-10 column. The desalted extract was purified on 5 mL-HiTrapQ HP column (GE Healthcare) equilibrated with Q start buffer using AKTA –FPLC system (Fast Protein Liquid Chromatography) and eluted with Q elution buffer (50 mM Tris-HCl pH 7.9 + 1 M NaCl) over 15 column volumes of increasing NaCl gradient (see typical elution profiles; Fig. S5). The active fractions eluted between 170 and 290 mM NaCl and were pale yellow in color. The active fractions (based on the chemiluminescence assay; see below) were pooled together into two separate stocks to be run on 120 mL Superdex75 column equilibrated with either 50 mM Tris-HCl pH 7.9 or ultra pure water. The active superdex fraction eluted in water were concentrated by vacuum centrifugation and then separated on SDS-PAGE. The gels were either stained with Coomassie Blue (0.1% Coomassie in 1% acetic acid and 40% methanol) or InstantBlue Coomassie (Expedeon, San Diego, CA). The protein bands (≈12, ≈19 and ≈25 kD) were excised, digested with trypsin and analyzed by LC-MS/MS at Proteomics Core Facility, University of California Davis (Davis, CA). The internal and N-terminal sequences generated were used to search for sequence match in the NCBI database, and to design primers for obtaining full protein sequence. The protein band showing sequences similarity to protein involved with iron biochemistry kept our particular attention.

### Total RNA isolation, cDNA synthesis, cloning, sequencing, subcloning and expression in *E. coli*

The aliform parapodia tissue was excised from the worm (Fig. 1A) and stored in RNA Later solution at 4 °C until the extraction. The total RNA was isolated using TRIzol Reagent and PureLink RNA isolation kit (Invitrogen) according to the manufacturer’s instructions. The first strand cDNA was synthesized from 320 ng total RNA using 18mer oligo-dT (IDT) and M-MLV RT. A full-length cDNA was amplified by PCR using Native Taq DNA polymerase (Invitrogen) and primers 5′-ATG GCC CAG ACH CAG CCN CG-3′ (forward) and 5′-TTA GCT GCT CAG GCT CTC CTT-3′ (reverse). The PCR was programmed as follow: initial denaturation at 95 °C for 2 min, then 35 cycles of 94 °C for 40 s, cooling at 46.4 °C for 1 min, and 72 °C for 1 min followed by a final extension at 72 °C for 10 min. A 519 bp PCR fragment was purified from agarose gel using Zymoclean Gel DNA Recovery kit (Zymo Research) and cloned into PCR-4 TOPO vector (Invitrogen) and transformed in One Shot TOP-10 competent cells (Invitrogen). The construct was verified by DNA sequencing. The cloned DNA insert was PCR amplified using high fidelity Pfx polymerase (Invitrogen) and primers 5′-CACC ATG GCC CAG ACT CAG CCG CG-3′ (forward) and 5′-TTA GCT GCT CAG GCT CTC CTT-3′ (reverse) PCR program 94 °C for 3 min then 30 cycles of 94 °C for 15 s, 60 °C for 30 s, 68 °C for 1 min followed by a final extension at 68 °C for 10 min. The amplified DNA, which showed best match to a ferritin-like protein, was gel purified and sub-cloned into pET 200 Directional TOPO expression vector (Invitrogen). The recombinant plasmid carrying ferritin-like cDNA was then transformed into BL21 star (DE3) *E*. *coli* cells (Invitrogen) to produce recombinant protein, worm ferritin (ChF). *E*. *coli* cells carrying recombinant were cultured overnight (16 h) at 37 °C in 20 mL LB containing 50 μg/mL kanamycin with shaking at 210 rpm. The overnight grown culture was transferred to a fresh 500 mL LB containing kanamycin and was grown until A_600 nm_ reached 0.7–0.8. To induce expression, Isopropyl β-D-thiogalactopyranoside was added at a final concentration of 1 mM. The culture was further incubated at 16 °C overnight under agitation at 250 rpm. The induced bacterial cells were pelleted down by centrifugation at 2,000 g for 20 min at 4 °C. The pellets were then stored at −80 °C until use.

### Purification of recombinant *Chaetopterus* Ferritin (ChF)

Bacterial pellets (1 g, w.w.) were suspended in 6 mL 1× Bugbuster Reagent (Novagen) mixed with 6 μL lysonase enzyme and incubated for 20 min at room temperature on a shaker. The lysate was cleared by centrifugation at 14,000 g for 15 min at 4 °C. The supernatant was desalted in a binding buffer containing 50 mM Sodium-Phosphate Buffer pH 7.7 and 0.03% Triton X-100 on a PD-10 column. The desalted extract was incubated with 1 mL cobalt-based TALON metal affinity resin (Clontech) pre-equilibrated with the same binding buffer at 4 °C on a rocker for 1 h. After incubation the sample-resin mixture was filtered through a disposable polypropylene column (Bio-Rad) kept on a collection tube to collect the filtrate containing unbound protein. The resin with bound protein in the column was washed with wash buffer (WB) containing 50 mM Sodium-Phosphate Buffer pH 7.7, 300 mM NaCl, 50 mM Imidazole and 0.03% Triton X-100. The recombinant HisTag protein was eluted in elution buffer (EB) containing 50 mM Sodium-Phosphate Buffer pH 7.7, 300 mM NaCl, 250–400 mM Imidazole and 0.03% Triton X-100. The eluted protein was treated with desalting buffer (DB) containing 50 mM Sodium-Phosphate pH 7.7 and 0.15 M NaCl to remove imidazole. The TALON purified recombinant protein was concentrated by ultrafiltration using 9 kD molecular weight cutoff membrane (Pierce, Thermo Scientific) and was subjected to Superdex200 column (16/60 GL) equilibrated with DB buffer for molecular weight estimation. The samples were stored at 4 °C until use. We used reference standards from Bio-Rad as well as GE Healthcare to estimate molecular weights for the proteins eluted from the size-exclusion chromatography. The standards included Thyroglobulin (670 kD), Υ-globulin (158 kD), Conalbumin (75 kD), Ovalbum (44 kD), Myoglobin (17 kD), Vitamin B12 (1.35 kD). The expression of recombinant ferritin (ChF) was ultimately confirmed by LC-MS/MS.

### Samples preparation for LC-MS/MS and protein quantification

The samples were desalted in water using 0.5 mL Zeba spin desalting column (Thermo Scientific) for use in SDS-PAGE for mass spectrometry analysis and protein quantification using A_280 _nm (E 1%) by Nanodrop.

### Chemiluminescence assay

Luminescence was measured using the Sirius luminometer described earlier. Light emission from protein samples in 50 mM Tris-HCl pH 7.9 in a final reaction volume of 500 μL was followed for 90 s, which included three consecutive treatments: background (0–30 s), after injection of 10 mM sodium persulfate (30–40 s), and after injection of 12% H_2_O_2_ (40–90 s). The total amount of light produced per treatment was calculated by integration over the respective time of each treatment. However, for simplification purposes in the data presented here, the chemical treatments were combined together, thus integrating 60 s (from 30 s to 90 s) of the assay (thus in RLU/min). Corresponding buffer was used as a control, which was subtracted from the treatments for better assessment of actual light produced by the samples. In this assay, light production was indicative of the presence of photoproteins and/or protein-bound chromophores.

### Effect of trace elements on light production from the weakly viscous phase of the mucus

Trace elements known to interfere (positively or negatively) with ferritin function were tested for their effect on light production. A total of 14 elements were tested, all at the same concentration of 20 mM in ASW, or MilliQ water when solubility issues occurred. Solutions were made fresh when indicated for some elements showing oxidation/precipitation. For light production analysis, an aliquot (50 μL) of the supernatant (mucus weakly viscous phase) was recorded for light production for 20 s (spontaneous light) after when the element solution was injected (v/v) and the light production recorded for an additional 20 s. We tried as much as possible to run one replicate of all element treatments and controls (injection of MilliQ or ASW) from the same supernatant, to decrease difference amongst elements due to inherent individual differences in mucus light intensity and/or viscosity, as reported earlier^7^. The experiments were repeated three to five times per element, each time using a different worm. For statistical analysis of the element effect, the total amount of light produced after element injection (sum of RLU/s from 20.2 s to 40.0 s) was expressed relative to the spontaneous amount of light (from 0.2 s to 20.0 s). Analysis of Variance (single factor ANOVA) and post-hoc multiple comparison of means (Fisher’s PLSD) were used to test significance of differences between effects of trace elements on light production compare to their specific control. Data were log(x + 1) transformed to comply with heteroscedasticity^62^.

### Ferroxidase activity assay for ChF

The ferroxidase activity was measured for purified recombinant protein by an end point assay^63^ using a chromogen Ferene S (3-(2-pyridyl)-5,6-bis(2-[5-furylsulfonic acid)-1-2,4-triazine). Ferene S forms a blue colored complex with soluble Fe^2+^ but not with Fe^3+^, which was read by absorbance at 562 nm using the Spectramax M2 spectrophotometer. Here, in order to preserve protein integrity (and their optimal biochemical function), slight modifications to the original assay buffer components included the no-use of thio-urea and chloroform. Also, a freshly prepared solution of FeSO_4_ in MilliQ water was used every time and stored in an airtight bottle to prevent auto-oxidation. A standard curve was prepared with varying concentration of FeSO_4_ solutions, which was shown to have 99.7% linearity down to 2 μM, and starting to show more variability above 40 μM. The assay was carried out three separate times (using different batches of recombinant proteins) in Sodium-Acetate buffer, pH 6.0 at 0.2 M final concentration in a 250 μL total reaction volume, after incubation of the reagents at 37 °C for 3–5 min. The purified recombinant protein was desalted in Sodium-Acetate buffer with 0.1 M NaCl and 10% glycerol before use, of which an aliquot of 50 μL was used for the affinity assays. The ferroxidase activity was expressed as change in substrate (Fe^2+^) concentration per minute per milligram of recombinant protein in an assay containing 33 μM FeSO_4_ incubated at 37 °C for 3 min, which was materialized by a decrease in intensity of blue color from the sample. By default the conventional enzymatic kinetics parameters V_max_ and K_m_ were determined by the Michaelis-Menten model, although we also used a Gompertz model (double rate model) for refinement since the light production seems to involve two competing reactions, between the luminous reagents and an inhibitor of the light production^8^.

### Iron quantification

The ferrozine-based colorimetric assay was used to quantify the amount of Fe^2+^ in the mucus (pellet as well as supernatant), following the recommended protocol^64^. The mucus samples were collected from six worms, and the samples prepared for the assay for which absorbance at 562 nm was measured with a Spectramax M2 spectrophotometer (Molecular Devices, Carlsbad, CA). A calibration curve with FeCl_2_ was first established (with R^2^ = 0.9966) and used to calculate the Fe^2+^ concentration in the sample^64^. In addition, the total amount of iron (Fe_tot_) was measured from the same samples of mucus (pellet as well as supernatant) using an Optima 3000 XL Inductively Coupled Plasma Spectrometry (ICP-OES, Perkin–Elmer, Waltham, MA). This was done by acidifying a known amount of the dried mucus with 200 μL of 70% nitric acid, which was then diluted to 5% with MilliQ water to perform the elements analysis with the ICP, as routinely done in our laboratory. The amount of iron was also measured from different body parts of worms. Body parts (Fig. 1A) included Head (H), Aliform Parapodia (AP), Black Sacks (BS), Mid/Gut (MG), White Sacks (WS), Cup-Shaped Parapodia (CP), and Tail (T), which were collected from eight freshly collected worms. After collection, each body part was oven dried, digested with nitric acid, and then diluted with MilliQ water before analysis. For each step of the process, the weight was recorded for dilution calculation, as routinely done in our laboratory^65^. Iron concentration data (Fe^2+^ and Fe_total_) were plotted for both the pellet and supernatant as a function of the initial amount of light recorded from the mucus sample. Plots were fit with Power regressions to help visualize trends.

### Carbohydrate and total protein analyses

Carbohydrate analysis was done by GC-MS at the UC San Diego Glycobiotechnology Core. Total protein was analyzed using Bio-Rad Rc-Dc protein assay kit.

### Materials and softwares

Oligonucleotides primers were from IDT (San Diego, CA). The purification was performed using an FPLC system (GE Healthcare, Pittsburgh PA) attached to a fraction collector and UV-VIS detector set at 280 nm (for general protein absorbance) and 360 nm (for ferritin specific absorbance^13^,^14^, and also checked here using horse ferritin; Sigma CAS #9007-73-2). Chromatography columns and Gel filtration standards kits were from GE Healthcare but also Bio-Rad (Hercules CA). TRIzol reagent and total RNA isolation kit were purchased from Invitrogen (Carlsbad, CA). All chemicals and reagents were molecular biology and HPLC grade. All calculations were done in Excel (Microsoft Inc.) while graphs were done with Deltagraph (RedRock Inc.) and statistics done with Statview^®^ 5.0 (SAS Institute Inc.).

### Availability of supporting data

The data set (*Chaetopterus* ferritin sequence) supporting the results of this article is available in GenBank repository [Nucleotide accession number KF471029 and protein accession number AIF79765 at http://www.ncbi.nlm.nih.gov/protein/665848569].

## Additional Information

**How to cite this article**: Rawat, R. and Deheyn, D. D. Evidence that ferritin is associated with light production in the mucus of the marine worm *Chaetopterus*. *Sci. Rep*. **6**, 36854; doi: 10.1038/srep36854 (2016).

**Publisher's note:** Springer Nature remains neutral with regard to jurisdictional claims in published maps and institutional affiliations.

## Supplementary Material

Supplementary Information

## Figures and Tables

**Table 1:** Analyses of monosaccharides (in %) and concentration (μg/μL) of total carbohydrates and proteins present in mucus from different body parts of the *Chaetopterus* worm.

Monosaccharide	Head	Body Parts		Tail
		Aliform parapodia	Cup-shaped parapodia	
Arabinose	3.97	1.05	0.00	0.41
Ribose	9.78	5.73	5.65	8.39
Rhamnose	0.00	1.14	0.00	0.00
Fucose	6.23	7.01	4.81	2.64
Xylose	0.00	3.37	3.88	0.89
Mannose	3.95	5.77	3.45	3.86
Galactose	24.2	21.63	44.37	17.83
Glucose	35.5	36.43	8.54	50.42
N-acetyl-D-glucosamine	10.09	12.96	22.77	10.14
N-acetyl-D-galactosamine	6.28	4.91	6.53	5.42
**Carbohydrates**	0.24	1.06	0.23	1.9
**Proteins**	471	1,620	1,203	2,522

**Table 2:** Significant ferritin peptides identified in a 19 kD protein band excised from SDS-PAGE and analyzed by tandem LC-MS/MS.

Ferritin peptide hits	Peptide identification probability	Homologs in polychaete species
DEWGTGLDAMQVALALEK	99.7%	*Pectinaria gouldii* [GenBank FJ416376]
IVLQNIQKPER	99.7%	*Alvinella pompejana* [GenBank GO176250]
NVNQSLLDLHK	99.7%	*Pectinaria koreni* [GenBank FR766789]
		*Capitella teleta* [GenBank EY608890]

**Table 3:** Concentration values (mean ± SD, minimum and maximum, in dry weight) in the mucus pellet and supernatant of Fe_tot_ (measured by ICP-OES) and Fe^2+^ (measured by the colorimetric ferrozine assay).

	Fe_tot_ (×10^−3 ^mg/g d.w.)		Fe^2+^ (×10^−3 ^mg/g d.w.)	
	Mean ± SD	Min.–Max.	Mean ± SD	Min.–Max.
Pellet	2.40 ± 2.48	0.271–6.82	0.195 ± 0.171	0.0489–0.490
Supernatant	0.395 ± 0.377	0.0754–1.02	0.0439 ± 0.0559	0.00134–0.138
