# Supplementary material for: Evidence that ferritin is associated with light production in the mucus of the marine worm Chaetopterus
Source: Sci Rep. 2016 Nov 10;6:36854. doi: 10.1038/srep36854 (PMC5103273; doi:10.1038/srep36854)
Supplement: Supplementary Information [file srep36854-s1.pdf]

Supplementary information

Evidence that ferritin is associated with light production in the mucus of the marine worm *Chaetopterus*

Renu Rawat and Dimitri D. Deheyn

Supplementary figures

Figure S1. Relationship between carbohydrate and protein concentration in *Chaetopterus* suggest mucus proteins are glycosylated.

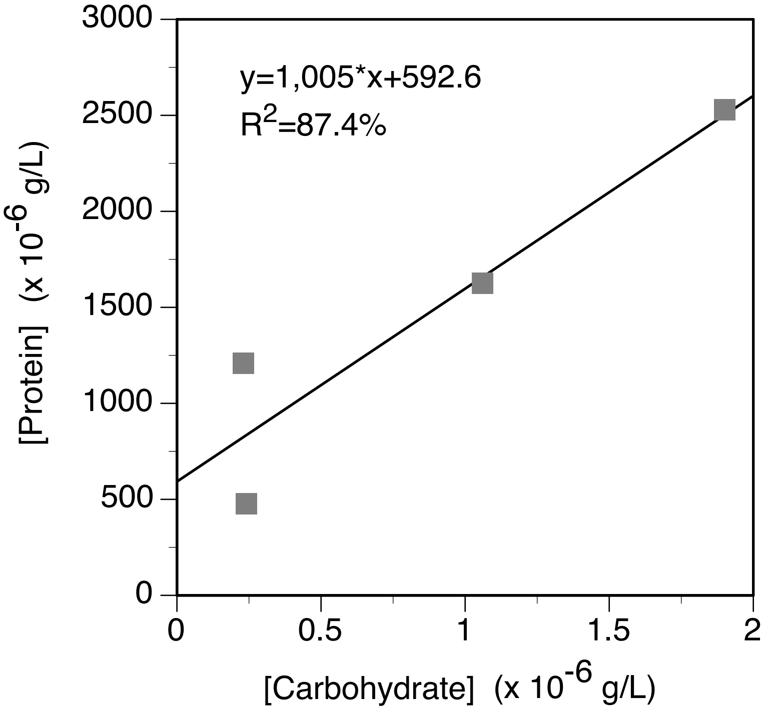

Figure S2. Superdex elutriate fractions (S1 to S10) obtained from FPLC partial purification process still have luminescence activity (CL activity with H_2O_2; see main text), and contain multiple proteins.

A. Elutriate fractions analyzed for CL, A_{280nm} (protein absorbance) and A_{360nm} (ferritin absorbance) show a peak of CL activity and absorbance for S5-S8. S5 shows low amount of proteins (as observed in panel B), yet a large CL activity. Data shown in relative scale (see Fig. 3 in main text for absolute scales).

B. SDS-PAGE gel of S5-S10 fractions (and subsequent fractions that showed no activity). Samples were separated by SDS-PAGE on 4-20 % Mini-Protean TGX gel and stained with Coomassie Blue. Protein ladder is Amersham ECL Rainbow Marker, High range (product # RPN756E). Three fine horizontal lanes were used for landmark positioning purpose only.

C. Closer view of the area of interest in the gel for lanes S6-S8, with the protein marker. Lanes show three major protein bands located around 12, 17, and 24kD markers. The one band around 17kD was identified as the worm ferritin (which in fact is 19kD). Work is in progress for bands ≈12kD and ≈25kD, but also ≈38kD.

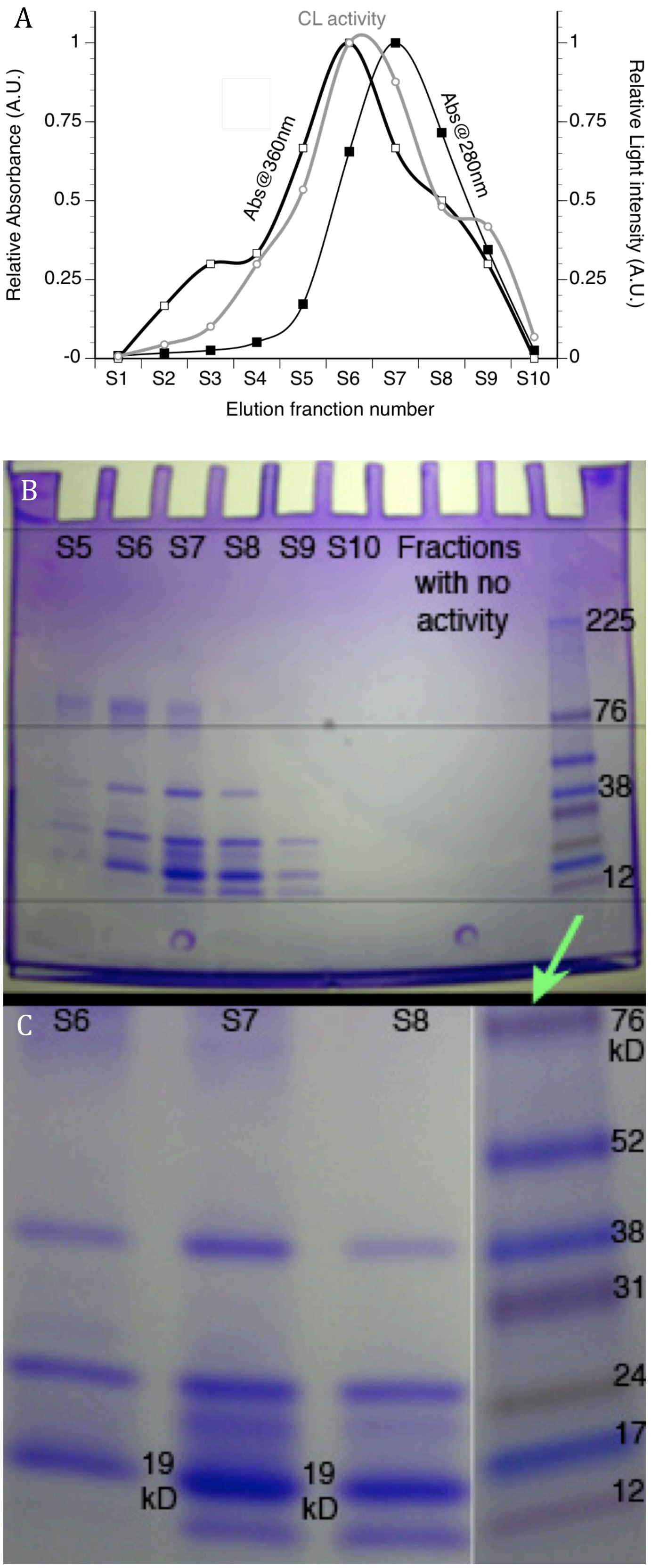

Figure S3. Ethidium bromide stained 1.2 % agarose gel showing PCR amplification of ferritin DNA from *Chaetopterus*. Lane 1 shows 519 bp DNA fragment amplified using cDNA generated by RT-PCR as a template. Lane 2 shows a 100 bp step DNA ladder (Invitrogen).

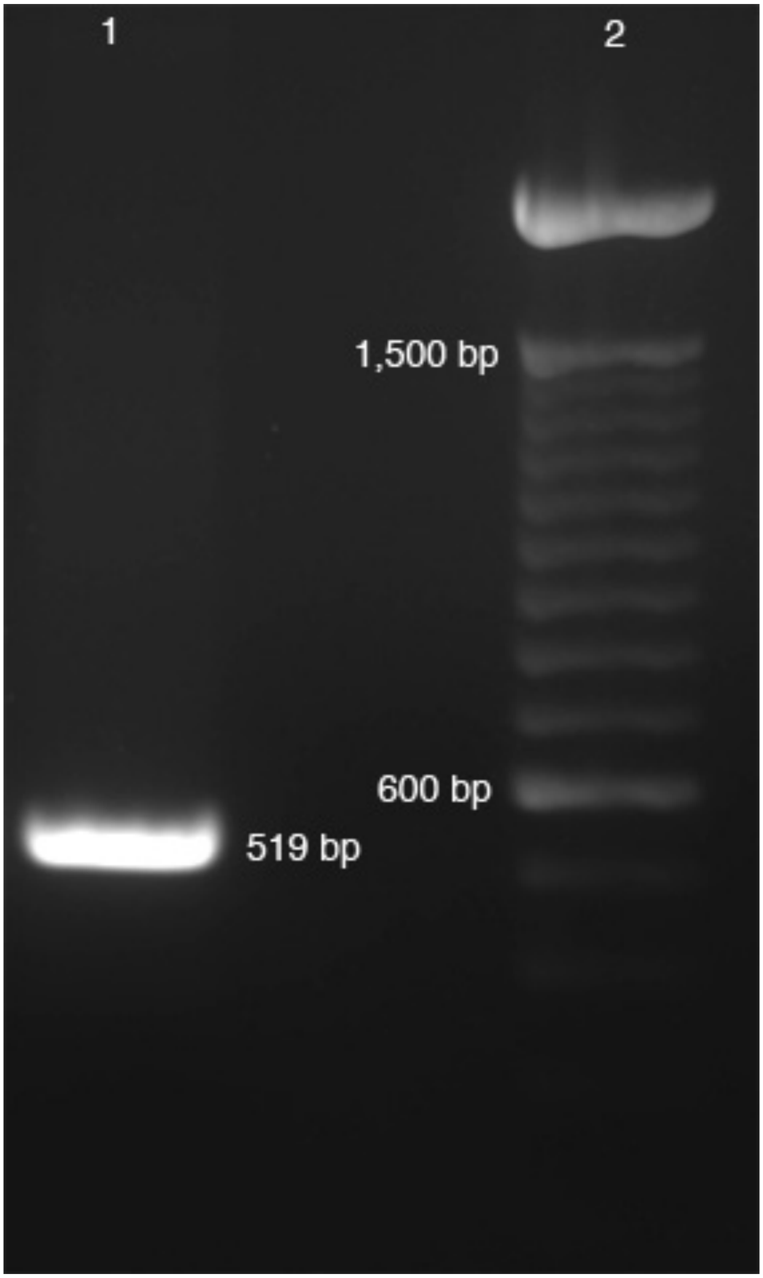

Figure S4. Iron concentrations (mg/g, d.w) for different body parts of *Chaetopterus*. Error bars represent deciles (10th, 90th percentiles), boxes represent quartiles (25th, 50th, 75th percentiles), and the filled squares represent the means. Legend: Head (H), Aliform Parapodia (AP), Black Sacks (BS), Mid/Gut (MG), White Sacks (WS), Cup-Shaped Parapodia (CP), and Tail (T).

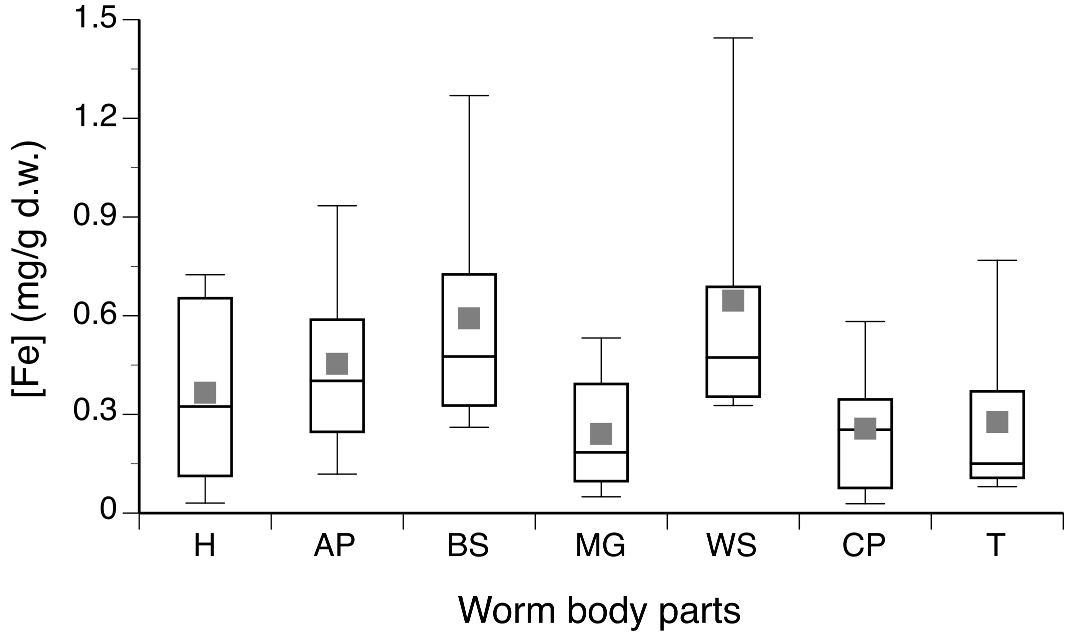

Figure S5: Two representative elution profiles made with an AKTA –FPLC system (Fast Protein Liquid Chromatography) of desalted extracts from the luminous mucus of the worm, using HiTrapQ HP column (GE Healthcare) equilibrated with Q start buffer and eluted with Q elution buffer (50 mM Tris-HCl pH 7.9 + 1 M NaCl). The eluted fractions were tested for bioluminescence capacity with H_2O_2 on a Sirius luminometer (Berthold Detection Systems, Germany).

Elution profile #1:

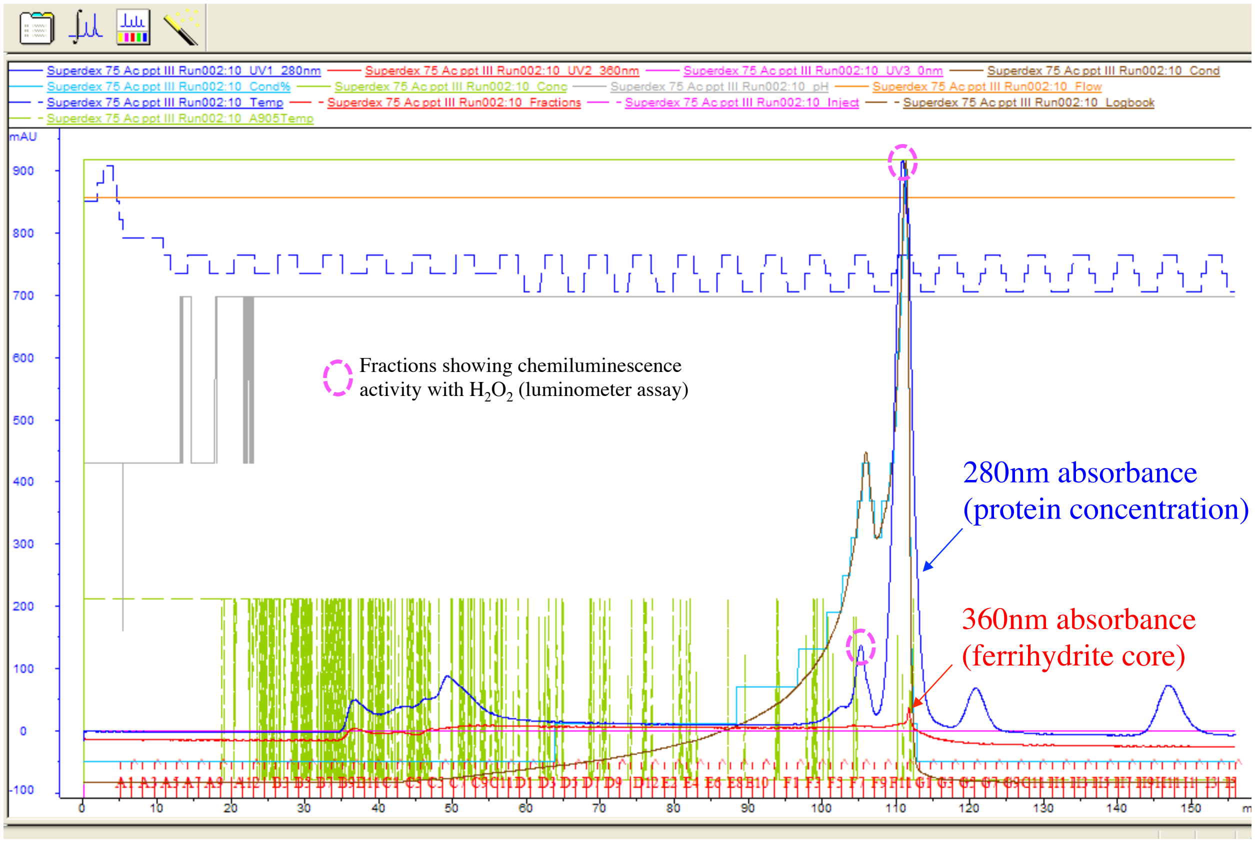

Elution profile #2:

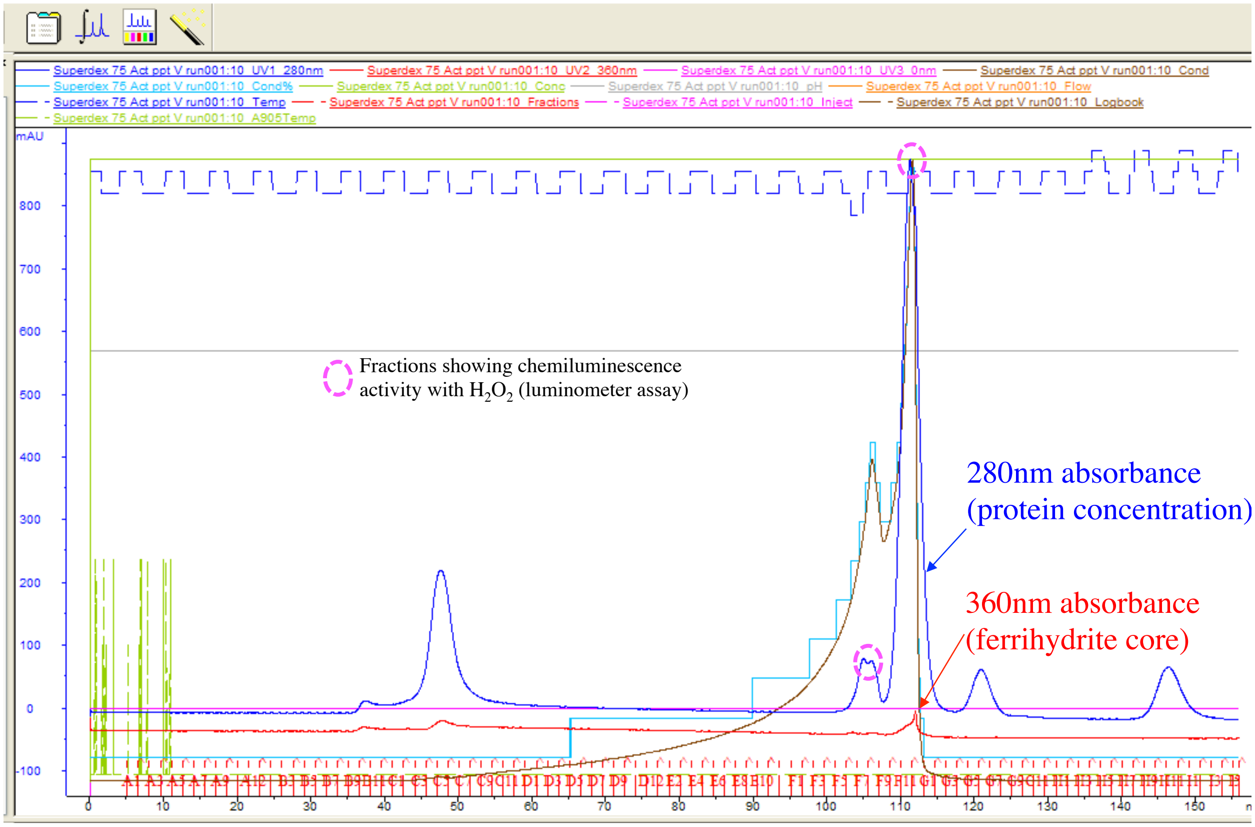

Supplementary table

Table S1. Chemiluminescence activity assay (CL) integrated over the 60s of chemical treatments (RLU) during the partial purification process of the mucus. Fold purification was represents CL activity for each step relative to the crude extract, while % yield is based on the protein amounts.

**Purification Steps**	**Total Volume** (mL)	**Total Protein** (mg)	**CL Activity** (Total volume)	**CL Activity** (Per protein)	**Fold Purifi-cation**	**% Yield**
(1) Crude Extract	30.0	150.0	210×10^6	1.4×10^6	1.0	100
(2) Desalted Extract PD-10 column	45.0	58.0	135×10^6	2.4×10^6	1.7	65.7
(3) HiTrap Q Pooled fractions	17.5	12.25	32×10^6	2.6×10^6	1.9	15.2
(4a) Ultrafiltration Retentate	0.9	3.51	4.0×10^6	1.1×10^6	0.8	1.9
(4b) Ultrafiltration Flow through	3.0	0.30	2.4×10^6	8.0×10^6	5.7	1.1
(5) Superdex 75 pooled Fractions	6.0	2.60	1.2×10^6	0.5×10^6	0.4	0.6
